# Effects of vitamin D combined with pioglitazone hydrochloride on bone mineral density and bone metabolism in Type 2 diabetic nephropathy

**DOI:** 10.1042/BSR20160544

**Published:** 2017-03-27

**Authors:** Ling-Xu Wang, Na Wang, Qing-Li Xu, Wei Yan, Li Dong, Bao-Lin Li

**Affiliations:** 1 Department of Clinical Laboratory, Zhengzhou Central Hospital Affiliated to Zhengzhou University, Zhengzhou 450007, P.R. China; 2 Department of Gynecology and Obstetrics, Women and Infants Hospital of Zhengzhou, Zhengzhou 450012, P.R. China; 3 Department of Anesthesiology, Zhengzhou Central Hospital Affiliated to Zhengzhou University, Zhengzhou 450007, P.R. China

**Keywords:** Bone mineral density, Bone metabolism, Pioglitazone hydrochloride, Type 2 diabetic nephropathy, Vitamin D

## Abstract

The study aims to investigate the effect of vitamin D (VD) combined with pioglitazone hydrochloride (PIO) on bone mineral density (BMD) and bone metabolism in patients with Type 2 diabetic nephropathy (T2DN). T2DN patients were selected and assigned into mild, moderate, and severe groups. In each group, three therapy regimens (VD, PIO, and VD plus PIO) were administered. X-ray absorptiometry was used to measure BMD. Intact parathyroid hormone (iPTH) and 25-hydroxyvitamin D3 (25-OH-VD3) were measured by chemiluminescence meter. ELISA was applied to detect levels of osteoprotegerin (OPG), bone gla protein (BGP), C-terminal telopeptides of type I collagen (β-CTX), procollagen type I N-propeptide (PINP), pyridinoline (Pyr), and deoxypyridinoline (D-Pyr). Compared with the mild group, T2DN patients in the moderate and severe groups had longer course of disease and higher levels of total cholesterol (TC), triglyceride (TG), serum phosphorus, fasting plasma glucose (FPG), glycosylated hemoglobin (HbAlc) and creatine (Cr), and lower blood calcium. The BMD in different parts increased among the mild, moderate, and severe groups, and the highest BMD was found after VD plus PIO treatment. OPG, iPTH, BGP, β-CTX, Pyr/Cr, and D-Pyr/Cr levels were reduced, while 25-OH-VD3 and PINP levels were elevated among three groups after different treatments, and the most obvious change was observed after VD plus PIO treatment. Our findings indicate that VD combined with PIO may be more effective in improving BMD and bone metabolism than VD or PIO alone in the treatment of T2DN patients, especially for T2DN patients with mild disease.

## Introduction

Type 2 diabetes mellitus (T2DM) is the most common type of diabetes, with a proportion of 90% in diagnosed DM cases globally, and is featured by insulin resistance and insulin secretion disorders [1]. It is noted that approximately 40% DM (including T1DM and T2DM) develops from diabetic nephropathy (DN) [2]. Type 2 diabetic nephropathy (T2DN) is a severe vascular complication of DM and is recognized as the principle cause of chronic kidney disease and death in DM patients [3]. T2DM evolution to T2DN originally begins from a slight increase in urinary albumin excretion (micro-albuminuria) to macro-albuminuria with higher and higher glomerular filtration rate (GFR), and eventually deteriorates into advanced renal disease that is pathologically characterized by glomerulosclerosis and tubulointerstitial fibrosis [4]. To the best of our knowledge, kidney is closely linked with changes in bone and mineral metabolism, and patients with nephrotic syndrome are found to have a predisposition to bone disease such as osteoporosis, which considerably results from metabolic imbalances of calcium and vitamin D (VD) [5]. In recent years, VD and pioglitazone hydrochloride (PIO) have become a new and popular research field, as impact factors of DM [6].

The typical role of VD is to regulate calcium and phosphorus metabolism as well as promote cell growth and differentiation [7]. A previous study has suggested that VD intake may reduce the risk of suffering from DM and serum 25-hydroxyvitamin D3 (25-OH-VD3) >95%, an active form of VD, is proved to be negatively associated with morbidity of T2DM [8]. In addition, VD plays a critical role in establishment and maintenance of bone health [9]. PIO is defined as a peroxisome proliferator-activated receptor-γ (PPAR-γ) agonist that is implicated in glucose metabolism by ameliorating insulin sensitivity and also triggers adipocyte differentiation [10]. Also, PIO is shown to not only possess renal protective effect as well as lower glucose [11], but also be able to improve endothelial function in patients with T2DM [11]. There is a study noting that DN along with insufficiency of insulin or inhibited insulin activity is the significant reason for decreased bone density and higher incidence of osteoporosis in elderly male patients with T2DM [12], indicating that bone mineral density (BMD) and alterations in bone metabolism are associated with DN. Thus, the present study is designed to investigate the effects of VD combined with PIO on BMD and bone metabolism in patients with T2DN.

## Materials and methods

### Ethics statement

The study has been approved by the Ethics Committee of Zhengzhou Central Hospital Affiliated to Zhengzhou University and all the patients had signed informed consent without exception.

### Study subjects

From July 2012 to January 2015, a total of 251 T2DN patients in the Zhengzhou Central Hospital Affiliated to Zhengzhou University were randomly selected. All patients were diagnosed according to the diagnostic criteria of diabetes released by American Diabetes Association (ADA) in 2007: fasting blood glucose (FBG) ≥7.0 mmol/l; hemoglobin A1C (HbA1c) ≥6.5%, blood glucose ≥11.1 mmol/l after oral glucose tolerance test (OGTT) 2 h later; or the random blood glucose of research subjects with typical hyperglycemia or hyperglycemic crisis ≥11.1 mmol/l; if no specific hyperglycemia was detected, FBG, HbA1c, and blood glucose of OGTT were measured repeatedly after 2 h for verification. The age, gender, height, weight, waistline, and hipline of the selected patients were recorded, and their disease courses were collected, finally their body mass index (BMI) = weight (kilograms)/height (meters) was calculated. Among 251 patients, 144 were males and 107 were females, with the age of 27.2–79 years and the mean age of 54.26 ± 8.15 years. And their disease course was from 0.5 to 22.8 years, with a mean of 7.59 ± 3.57 years. The inclusion criteria was as follows: patients without chronic hepatic diseases, endocrine metabolic diseases exampled by hyperthyroidism; patients without calcium and phosphorus metabolic disorders such as osteoarticular diseases and bone metastases; patients without serious systemic diseases, ketoacidosis or history of long-term immobilization; patients without medicine-taking history of hormones, VD3, and calcium supplement. Female patients without menses for more than 10 years were selected.

On the basis of DN staging, 251 patients were arranged into three groups according to the changes of GFR during DN course [13]: mild group (*n*=87, GFR ≥90 ml/min), moderate group (*n*=80, 60 ml/min ≤ GFR ≤ 90 ml/min), and severe group (*n*=84, GFR ≤90 ml/min). In each group, T2DN patients were treated with three therapy regimens (VD alone, PIO alone, and VD plus PIO). There were no differences in age and gender of patients in each group.

### Data collection

The height, weight, and BMI of T2DN patients were measured, and 7600-210 biochemical automatic analyzer produced by Hitachi Data System Corporation, Santa Clara, CA, U.S.A. was used to measure glycosylated hemoglobin (HbAlc), triglyceride (TG), total cholesterol (TC), low-density lipoprotein cholesterol (LDL-C), high-density lipoprotein cholesterol (HDL-C), and serum calcium, serum phosphorus, serum creatine (Cr). Fasting plasma glucose (FPG) was measured by glucose oxidase method.

### Therapy regimens

The same dosage of hypoglycemic was conducted in patients of VD therapy for the whole treatment. After the first 14 days, VD3 250 IU/d and elemental calcium 1200 mg/d (two pills of Caltrate D per day) were taken by the patients with VD alone treatment; one pill of PIO (30 mg/p, Jiangsu DeYuan Pharmaceuticals Co., Ltd, Jiangsu, China) was taken by the patients of PIO alone treatment before breakfast every morning; VD3 250 IU/d and elemental calcium 1200 mg/d (two pills of Caltrate D per day) plus one pill of PIO (30 mg/p, Jiangsu DeYuan Pharmaceuticals Co., Ltd, Jiangsu, China) were taken by the patients of VD plus PIO therapy before breakfast every morning. Meanwhile, the patients in each group were subject to lifestyle interventions such as energy intake and proper exercise. In the 4th and 8th week, the BMI of each patient was obtained. Every two weeks, follow-up was conducted and then liver function was tested to make sure that included patients were free from serious liver lesion, edema, and heart failure. In the 12th week after intervention, we tested those patients for the BMI, 25-OH-VD, TG, FPG, and fasting insulin (FINS). Medicines of antiplatelet, anti-freezing, vascular dilation, lipid-decreasing, and angiotensin-converting enzyme inhibitors (ACEI) were forbidden during the whole treatment.

### Bone densitometry

Dual-Energy X-ray Absorptiometry (Hologic Inc., Bedford MA, U.S.A.) was used for bone densitometry. After treatment, every subject was examined on supine position to get the BMD (g/cm^2^) of L2∼4, neck, G.T, hip joint, Ward’s triangle, and focile.

### Measurement of bone metabolism

Serum samples from the patients after treatment with empty stomach were collected and then were stored at 2–8°C. A Roche E-170 Chemiluminescence Meter was adopted to test intact parathyroid hormone (iPTH) and 25-OH-VD3. ELISA was used to measure osteoprotegerin (OPG), bone gla protein (BGP), C-terminal telopeptides of type I collagen (β-CTX), procollagen type I N-propeptide (PINP), pyridinoline (Pyr), and deoxypyridinoline (D-Pyr). The ratio of Pyr/Cr and D-Pyr/Cr was calculated. Carbonate-coated buffer (0.05 M, pH 9) was used to dilute antibody to protein concentration of 1–10 μg/ml, and 0.1 ml of antibody was added into every reaction well supplemented with polystyrene and maintained at 4°C for whole night. After throwing away solution, each well was washed three times. The sample (0.1 ml) under certain dilution was added into the wells treated after above process (the blank well is a well without sample and ELISA reagent). Following incubation at 37°C for 1 h, the wells were washed and then added with fresh diluted ELISA antibody (0.1 ml), incubated at 37°C for half an hour. Subsequently, coloration was detected at 37°C for 15 min. With the addition of stop buffer to each well, optical density (OD) value was detected with the blank well as the zero well at wavelength of 450 nm.

### Statistical analysis

Statistical package for the social sciences (SPSS) software 20.0 (SPSS Inc. IBM, Chicago, IL, U.S.A.) was used for statistical analysis. Measurement data are presented as mean ± standard deviation; homogeneity of variance was examined before comparison between pre- and post-treatment in a group and between sample mean of two groups. *t*-test was used for homogeneity of variance and rank test for heterogeneity of variance. ANOVA was performed for comparison among multiple groups. Spearman analysis was conducted for analysis of OPG and T2DN in different severity, and its correlation between other indices was confirmed using Pearson correlation analysis. *P*<0.05 represented significant difference.

## Results

### Baseline characteristics of T2DN patients

The data in Table 1 showed a comparison of baseline characteristics between 251 T2DN patients of mild, moderate, and severe groups. There was no significant difference of age, gender, BMI, and LDL-C among three groups (*P*>0.05). Compared with those in the mild group, T2DN patients in the moderate and severe groups had longer course of disease and higher levels of TC, TG, HDL-C, serum phosphorus, FPG, HbAlc, and Cr, while blood calcium was lower (all *P*<0.05). The course of disease, TC, serum phosphorus, FPG, HbAlc, and Cr were enhanced, but blood calcium was declined in the severe group as compared with the moderate group (all *P*<0.05).

**Table 1:** Baseline characteristics of patients with T2DN

Characteristic	Mild group (*n*=87)	Moderate group (*n*=80)	Severe group (*n*=84)
Age (years)	54.14 ± 8.10	53.82 ± 7.47	54.52 ± 8.56
Gender (male/female)	50/37	39/41	48/36
Disease course (years)	6.57 ± 2.25	7.90 ± 2.90*	11.17 ± 4.24^*†^
BMI (kg/m^2^)	24.50 ± 3.28	23.38 ± 2.87	23.64 ± 3.03
TC (mmol/l)	4.62 ± 0.83	4.95 ± 0.43*	5.45 ± 0.64^*†^
TG (mmol/l)	1.63 ± 0.45	1.72 ± 0.40*	1.73 ± 0.47*
HDL-C (mmol/l)	1.48 ± 0.40	1.63 ± 0.37*	1.66 ± 0.35*
LDL-C (mmol/l)	2.57 ± 0.66	2.54 ± 0.59	2.64 ± 0.62
Blood calcium (mmol/l)	2.07 ± 0.30	1.76 ± 0.11*	1.59 ± 0.62^*†^
Phosphorus (mmol/l)	0.95 ± 0.29	1.19 ± 0.19*	1.51 ± 0.33^*†^
FPG (mmol/l)	9.23 ± 0.86	9.82 ± 1.08*	10.73 ± 1.01^*†^
HbAlc (%)	7.35 ± 1.43	8.50 ± 1.65*	9.61 ± 2.00^*†^
Cr (μmol/l)	105.47 ± 22.75	163.19 ± 29.26*	220.92 ± 31.95^*†^

Note: *P<0.05 compared with the mild group; ^†^*P*<0.05 compared with the moderate group; Cr, serum creatine.

### Changes of bone mineral density of T2DN patients among three groups

**Figure 1:**
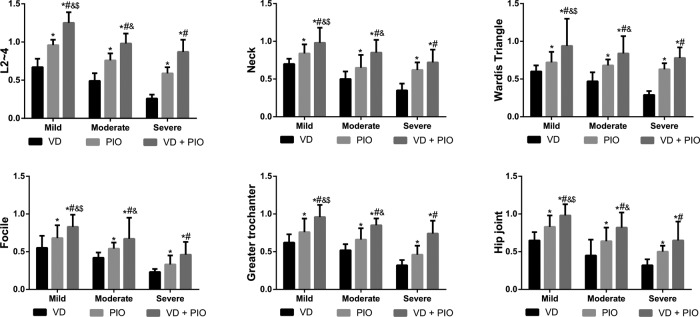
Comparisons of BMD of patients with T2DN among three groups Note: *P<0.05 compared with the VD group; ^#^*P*<0.05 compared with the PIO group; ^&^*P*<0.05 compared with the severe group; ^$^*P*<0.05 compared with moderate group; VD + PIO, vitamin D plus pioglitazone hydrochloride.

After VD alone, PIO alone, and VD plus PIO treatment, the BMD in different parts (L2∼4, neck, Ward’s triangle, focile, greater trochanter, and hip joint) was gradually increased among three groups, and the highest BMD was found after VD plus PIO treatment (all *P*<0.05). After VD plus PIO treatment, the BMD in the mild group was higher than that in the moderate group, and the BMD in the moderate group was higher than that in the severe group (all *P*<0.05, as shown in Table 2 and Figure 1).

**Table 2:** Comparisons of BMD of patients with T2DN among three groups

Group	BMD at different parts					
	L2∼4	Neck	Ward’s triangle	Focile	Greater trochanter	Hip joint
Mild group						
VD	0.67 ± 0.11	0.70 ± 0.07	0.60 ± 0.08	0.55 ± 0.16	0.62 ± 0.11	0.65 ± 0.11
PIO	0.96 ± 0.07*	0.84 ± 0.12*	0.72 ± 0.14*	0.68 ± 0.17*	0.76 ± 0.18*	0.83 ± 0.15*
VD + PIO	1.25 ± 0.14^*^^†‡^^§^	0.98 ± 0.20^*^^†‡^^§^	0.94 ± 0.36^*^^†‡§^	0.83 ± 0.16^*^^†‡^^§^	0.96 ± 0.16^*^^†‡^^§^	0.98 ± 0.15^*^^†^^‡^^§^
Moderate group						
VD	0.49 ± 0.10	0.50 ± 0.10	0.47 ± 0.12	0.42 ± 0.07	0.52 ± 0.08	0.45 ± 0.21
PIO	0.76 ± 0.09*	0.65 ± 0.17*	0.68 ± 0.08*	0.54 ± 0.08*	0.66 ± 0.15*	0.64 ± 0.18*
VD + PIO	0.98 ± 0.13^*†‡^	0.85 ± 0.17^*†‡^	0.84 ± 0.23^*†‡^	0.67 ± 0.28^*^^†^^‡^	0.85 ± 0.09^*^^†^^‡^	0.82 ± 0.20^*^^†^^‡^
Severe group						
VD	0.26 ± 0.05	0.35 ± 0.09	0.29 ± 0.05	0.23 ± 0.04	0.32 ± 0.07	0.32 ± 0.08
PIO	0.59 ± 0.08*	0.62 ± 0.10*	0.63 ± 0.08*	0.33 ± 0.12*	0.46 ± 0.12*	0.50 ± 0.08*
VD + PIO	0.87 ± 0.16^*†^	0.72 ± 0.17^*†^	0.78 ± 0.14^*†^	0.46 ± 0.17^*†^	0.74 ± 0.17^*†^	0.65 ± 0.25^*†^

Note: *P<0.05 compared with the VD group; ^†^*P*<0.05 compared with the PIO group; ^‡^*P*<0.05 compared with the severe group; ^§^*P*<0.05 compared with the moderate group.

### Comparisons of bone metabolism parameters of T2DN patients among three groups

**Figure 2:**
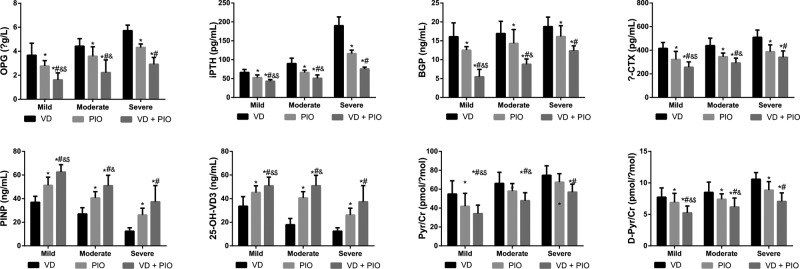
Comparisons of bone metabolism parameters of patients with T2DN among three groups Note: *P<0.05 compared with the VD group; ^#^*P*<0.05 compared with the PIO group; ^&^*P*<0.05 compared with the severe group; ^$^*P*<0.05 compared with the moderate group; VD + PIO, vitamin D plus pioglitazone hydrochloride.

The levels of OPG, iPTH, BGP, β-CTX, Pyr/Cr, and D-Pyr/Cr were reduced, while 25-OH-VD3 and PINP levels were elevated in T2DN patients among the three groups after VD alone, PIO alone, and VD plus PIO treatment (all *P*<0.05), the most obvious change was observed after VD plus PIO treatment. In comparison with the severe group, the levels of OPG, iPTH, BGP, β-CTX, Pyr/Cr, and D-Pyr/Cr were declined, but 25-OH-VD3 and PINP levels were increased in the mild and moderate groups (all *P*<0.05); while the levels of OPG, iPTH, BGP, β-CTX, Pyr/Cr, and D-Pyr/Cr were lower in the mild group than those in the moderate group (all *P*<0.05) (Table 3 and Figure 2).

**Table 3:** Comparisons of bone metabolism parameters of patients with T2DN among three groups

Group	OPG (μg/l)	iPTH (pg/ml)	BGP (ng/ml)	β-CTX (pg/ml)	PINP (ng/ml)	25-OH-VD3 (ng/ml)	Pyr/Cr (pmol/μmol)	D-Pyr/Cr (pmol/μmol)
Mild group								
VD	3.67 ± 1.00	66.39 ± 7.59	16.10 ± 3.67	415.71 ± 50.43	36.99 ± 4.96	33.66 ± 8.07	54.91 ± 14.01	7.73 ± 1.48
PIO	2.77 ± 0.45*	52.85 ± 6.82*	12.54 ± 0.92*	322.60 ± 67.47*	51.33 ± 6.90*	45.20 ± 5.69*	41.99 ± 13.64*	6.89 ± 1.45*
VD + PIO	1.62 ± 0.58^*^^†‡^^§^	43.28 ± 4.09^*^^†^^‡^^§^	5.48 ± 1.95^*^^†^^‡^^§^	256.03 ± 45.88^*^^†^^‡^^§^	62.59 ± 6.15^*†‡§^	50.82 ± 7.38^*^^†^^‡^^§^	34.18 ± 8.91^*^^†^^‡§^	5.24 ± 1.08^*^^†^^‡^^§^
Moderate group								
VD	4.42 ± 0.63	89.94 ± 14.16	16.92 ± 3.23	438.92 ± 63.58	27.06 ± 5.27	17.88 ± 4.84	66.12 ± 11.75	8.49 ± 1.64
PIO	3.59 ± 0.78*	66.45 ± 5.99*	14.33 ± 3.66*	345.50 ± 31.16*	40.62 ± 5.26*	27.01 ± 5.84*	58.02 ± 7.99*	7.40 ± 0.87*
VD + PIO	2.28 ± 1.05^*^^†^^‡^	50.91 ± 8.80^*^^†^^‡^	8.80 ± 1.39^*^^†^^‡^	292.12 ± 41.37^*†‡^	50.98 ± 8.80^*†‡^	42.77 ± 4.20*^†‡^	47.87 ± 8.46*^†^^‡^	6.18 ± 1.41*^†^^‡^
Severe group								
VD	5.72 ± 0.46	190.14 ± 23.02	18.77 ± 2.52	509.57 ± 62.30	12.43 ± 2.95	11.12 ± 3.34	74.74 ± 10.02	10.58 ± 1.05
PIO	4.32 ± 0.29*	116.28 ± 9.08*	16.14 ± 2.86*	388.53 ± 57.39*	26.14 ± 5.79*	20.33 ± 5.93*	67.42 ± 8.97*	8.85 ± 1.35*
VD + PIO	2.92 ± 0.56*^†^	75.49 ± 4.92*^†^	12.37 ± 1.31*^†^	341.30 ± 52.88*^†^	37.37 ± 13.67*^†^	36.57 ± 9.33*^†^	56.96 ± 8.44*^†^	7.06 ± 1.36*^†^

Note: *P<0.05 compared with the VD group; ^†^*P*<0.05 compared with the PIO group; ^‡^*P*<0.05 compared with the severe group; ^§^*P*<0.05 compared with the moderate group; VD + PIO, vitamin D plus pioglitazone hydrochloride.

### Correlations of OPG level with bone metabolism parameters of T2DN patients

**Figure 3:**
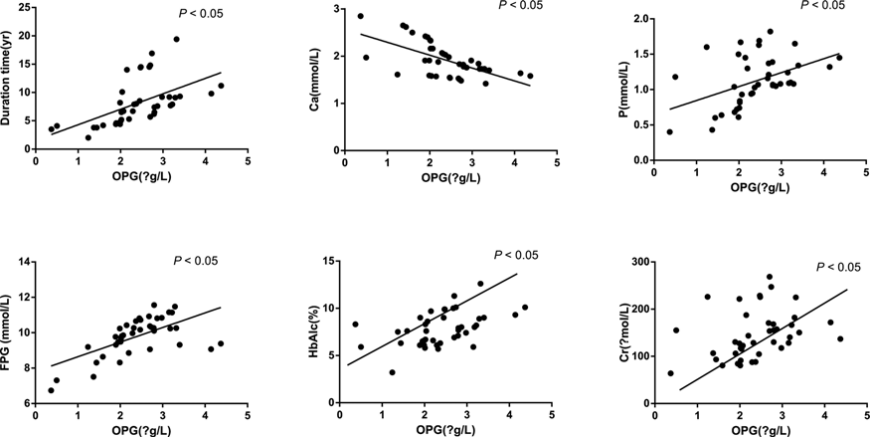
Correlations of OPG level with bone metabolism parameters of T2DN patients Note: Ca, serum calcium; P, serum phosphorus; Cr, serum creatine.

The Spearman analysis was adopted to analyze the correlations of OPG level with T2DN patients with different disease severity after VD plus PIO treatment, and the parameters including the course of disease, TC, blood calcium, serum phosphorus, FPG, HbAlc, and Cr of patients among the three groups were analyzed using the Pearson analysis. As shown in Table 4 and Figure 3, after VD plus PIO treatment, the OPG level in T2DN patients was positively correlated with different severity of the disease, course of disease, serum phosphorus, FPG, Cr, and HbA1c (all *P*<0.05), and negatively related to blood calcium (*P*<0.05), but it was not associated with TC level (*P*>0.05).

**Table 4:** Correlations of OPG level with bone metabolism parameters of T2DN patients

Indicator	OPG level	
	*r*	*P*
Disease severity of T2DN	0.597	<0.001
Disease course	0.378	<0.001
TC	0.158	0.157
Blood calcium	−0.365	<0.001
Serum phosphorus	0.455	<0.001
FPG	0.245	0.027
HbAlc	0.345	0.002
Serum creatine	0.506	<0.001

## Discussion

DN is accompanied by abnormal bone metabolism and easily leads to osteodystrophy and osteoporosis, which are serious complications of diabetes that appear in the skeletal system [14]. Importantly, increasing evidence reveal that the decreased BMD is due to reduced bone matrix synthesis that is caused by the considerable loss of calcium, phosphorus, and magnesium with hyperosmotic diuresis. Clinically, patients with diabetic osteoporosis would show severe pain and dysfunction, with high morbidity [12,15]. It is known that bisphosphonate is commonly used for bone protection, and Conley et al. [16] indicated that bisphosphonates is able to prevent bone loss in diabetes associated with mineral bone disease patients; however, Coe et al. [17] demonstrated that long term of bisphosphonate therapy would further suppressing bone formation in diabetic mice although it enhanced bone density. Currently, VD levels and balance are also demonstrated to be vital in bone protection [18]; however, current recommendations for VD intake are inadequate, particularly for populations at risk for suboptimal VD status, such as those with diabetes and kidney disease. Therefore, the present study is designed to investigate the effects of VD plus PIO on BMD and bone metabolism of T2DN patients.

One of the results demonstrates that BMD in patients with T2DN is increased after VD plus PIO treatment. VD and its analogs are supposed to prevent kidney damage in DN by inhibiting the renin–angiotensin system (RAS), which plays a central role in the regulation of blood pressure, electrolyte, and volume homeostasis [19–21]. Recently, evidence has been collected to suggest that VD may inhibit the production of transforming growth factor-β (TGF-β), which is a pro-fibrotic factor and plays a key role in diabetic glomerulosclerosis process and monocyte chemoattractant protein-1 (MCP-1), thus reducing the occurrence of glomerular sclerosis [22–25]. At the same time, VD, especially 25-OH-VD3, which regulates calcium, phosphorus metabolism, and bone metabolism balance, is an important class of steroid hormones [26,27]. Both Reid et al. [28] and Kogawa et al. [29] provide evidence showing that drugs of VD can effectively increase BMD in patients with osteoporosis due to the fact that VD preparations may stimulate the differentiation of bone cell precursors and inhibit the interaction between the front osteoblast and osteoclast precursor with bone resorption reduced. PIO belongs to thiazolidinedione derivatives (insulin sensitizer) and is an agonist of PPAR-γ. It can prevent kidney from damage through activating PPAR-γ, improving insulin sensitivity, and reducing urinary albumin excretion rate in patients with diabetes [30–32]. PPAR-γ stimulation can affect the regulation of the mesenchymal stem cells and stimulate differentiation into adipocytes especially in osteoblasts [33]. A previous study in healthy women showed that thiazolidinedione treatment was correlated with BMD and markers of bone formation [34]. Moreover, osteoporosis is associated with atherosclerosis in T2 diabetes, and PIO administration has been proved to be able to prevent atherosclerosis progression [35]. Similarly, Teramoto et al. [36] indicate that OPG is declined after rosiglitazone treatment in the T2DM. Several studies show that BMD is significantly higher in patients with Type 2 diabetes after combined intervention of VD and PIO, but the effect of PIO alone on BMD is not obvious [30,37].

Also, our study proves that the levels of OPG, iPTH, β-CTX, BGP, Pyr/Cr, and D-Pyr/Cr of the T2DN patients are dropped and the PINP levels and 25-OH-VD3 are elevated after VD plus PIO therapy. To the best of our knowledge, OPG is a tumor decoy receptor, and it can inhibit osteoclast formation, differentiation and survival, and induce its apoptosis, so OPG may participate in bone metabolism. While we may assume that the reason why diabetic OPG is elevated in serum is that OPG expression in renal tubular cells is significantly increased in the patients with DN [38]. PINP, as a biological response marker, an osteoblast-derived protein, may provide clinically useful information for managing patients with osteoporosis during teriparatide treatment [39]. Also, iPTH is a more specific and sensitive marker of bone formation [40]. Furthermore, when osteoclast activity is enhanced, β-CTX is released into the blood, thus β-CTX is believed to be a valuable indicator in bone resorption and formation [41]. Substantially, serum BGP plays an essential role in the regulation of bone and calcium metabolism, and blood concentration of BGP is supposed to reflect the activity of osteoblasts and regarded as a sensitive and specific marker [42]. Also, the binding of BGP and bone calcium depends on vitamin K and regulated by VD [43]. In addition, Nur et al. [44] have demonstrated that Pyr and D-Pyr are considered as collagen cross-links and their urinary concentrations have been identified as important markers of bone resorption, and the increase in urinary excretion of Pyr and D-Pyr leads to bone ischemia and subsequent necrosis caused by microvascular occlusion that induces bone degradation. Thus, we can conclude that the decrease in Pyr and D-Pyr levels may produce good effects on bone formation. In our study, compared with VD or PIO alone, the 25-OH-VD3 and PINP levels are significantly increased after VD plus PIO therapy in T2DN patients.

## Conclusion

In conclusion, our findings indicate that VD combined with PIO may be more effective in improving BMD and bone metabolism than VD or PIO alone in the treatment of T2DN patients, especially for T2DN patients with mild disease. Our study provides guidance for clinical rational drug use in the treatment of T2DN patients with different disease severity. However, the relevant mechanism of drug intervention had not yet proved. Therefore, further studies are needed to provide deep investigation to optimize the clinical treatment.

## Abbreviations

β-CTX: C-terminal telopeptides of type I collagen
25-OH-VD3: 25-hydroxyvitamin D3
BGP: bone gla protein
BMD: bone mineral density
BMI: body mass index
DM: diabetes mellitus
DN: diabetic nephropathy
D-Pyr: deoxypyridinoline
FBG: fasting blood glucose
FPG: fasting plasma glucose
GFR: glomerular filtration rate
GT: greater trochanter
HbAlc: glycosylated hemoglobin
HDL-C: high-density lipoprotein cholesterol
iPTH: intact parathyroid hormone
LDL-C: low-density lipoprotein cholesterol
OGTT: oral glucose tolerance test
OPG: osteoprotegerin
PINP: procollagen type I N-propeptide
PIO: pioglitazone hydrochloride
PPAR-γ: peroxisome proliferator-activated receptor-γ
Pyr: pyridinoline
TC: total cholesterol
T2DM: Type 2 diabetes mellitus
T2DN: Type 2 diabetic nephropathy
TG: triglyceride
VD: vitamin D
